# Superior temporal sulcus folding, functional network connectivity, and autistic-like traits in a non-clinical population

**DOI:** 10.1186/s13229-024-00623-3

**Published:** 2024-10-08

**Authors:** Igor Nenadić, Yvonne Schröder, Jonas Hoffmann, Ulrika Evermann, Julia-Katharina Pfarr, Aliénor Bergmann, Daniela Michelle Hohmann, Boris Keil, Ahmad Abu-Akel, Sanna Stroth, Inge Kamp-Becker, Andreas Jansen, Sarah Grezellschak, Tina Meller

**Affiliations:** 1 Cognitive Neuropsychiatry Lab, Department of Psychiatry and Psychotherapy, Philipps-Universität Marburg, Rudolf-Bultmann-Str. 8, 35037 Marburg, Germany; 2 Center for Mind, Brain, and Behavior (CMBB), University of Marburg, Justus Liebig University Gießen, and Technical University of Darmstadt, Hans-Meerwein-Straße 6, 35032 Marburg, Germany; 3 Marburg University Hospital – UKGM, Marburg, Germany; 4 Institute of Medical Physics and Radiation Protection, Department of Life Science Engineering, TH Mittelhessen University of Applied Sciences, Giessen, Germany; 5 LOEWE Research Cluster for Advanced Medical Physics in Imaging and Therapy (ADMIT), TH Mittelhessen University of Applied Sciences, 35390 Giessen, Germany; 6 Department of Diagnostic and Interventional Radiology, University Hospital Marburg, Philipps-Universität Marburg, Marburg, Germany; 7 School of Psychological Sciences, University of Haifa, Haifa, Israel; 8 The Haifa Brain and Behavior Hub (HBBH), University of Haifa, Haifa, Israel; 9 Department of Child and Adolescent Psychiatry and Psychotherapy, Philipps-Universität Marburg, Marburg, Germany; 10 BrainImaging Core Facility, School of Medicine, Philipps-Universität Marburg, Marburg, Germany; 11 LOEWE Center DYNAMIC, University of Marburg, Marburg, Germany

**Keywords:** Autism quotient (AQ), Autism spectrum disorder (ASD), Cortical surface complexity, Interpersonal, Subclinical

## Abstract

**Background:**

Autistic-like traits (ALT) are prevalent across the general population and might be linked to some facets of a broader autism spectrum disorder (ASD) phenotype. Recent studies suggest an association of these traits with both genetic and brain structural markers in non-autistic individuals, showing similar spatial location of findings observed in ASD and thus suggesting a potential neurobiological continuum.

**Methods:**

In this study, we first tested an association of ALTs (assessed with the AQ questionnaire) with cortical complexity, a cortical surface marker of early neurodevelopment, and then the association with disrupted functional connectivity. We analysed structural T1-weighted and resting-state functional MRI scans in 250 psychiatrically healthy individuals without a history of early developmental disorders, in a first step using the CAT12 toolbox for cortical complexity analysis and in a second step we used regional cortical complexity findings to apply the CONN toolbox for seed-based functional connectivity analysis.

**Results:**

Our findings show a significant negative correlation of both AQ total and AQ attention switching subscores with left superior temporal sulcus (STS) cortical folding complexity, with the former being significantly correlated with STS to left lateral occipital cortex connectivity, while the latter showed significant positive correlation of STS to left inferior/middle frontal gyrus connectivity (*n* = 233; all *p* < 0.05, FWE cluster-level corrected). Additional analyses also revealed a significant correlation of AQ attention to detail subscores with STS to left lateral occipital cortex connectivity.

**Limitations:**

Phenotyping might affect association results (e.g. choice of inventories); in addition, our study was limited to subclinical expressions of autistic-like traits.

**Conclusions:**

Our findings provide further evidence for biological correlates of ALT even in the absence of clinical ASD, while establishing a link between structural variation of early developmental origin and functional connectivity.

**Supplementary Information:**

The online version contains supplementary material available at 10.1186/s13229-024-00623-3.

## Introduction

Conceptualising psychiatric disorders as disease spectra or dimensions, rather than distinct disease categories, raises the question of dimensional biological correlates. As most psychiatric imaging studies rely on case-control-designs to identify group-level differences of the impact of a particular clinical phenotype, they might be less suited to capture variation related to subclinical phenotypes. For several neuropsychiatric disorders, there is now increasing evidence that such subclinical variation is indeed related to variation in brain structure or function – as shown in affective disorders [1] or psychosis spectrum markers [2, 3], and more recently also with autistic traits [4–7]. This motivates novel approaches to studying variation related to psychiatric conditions beyond the classical case-control design. In particular, by using dimensional markers such as quantifiable traits (e.g., obtained through behavioural assessments of self-report inventories), which have a biological relation to mechanisms implicated in the respective disease spectrum, one might use a correlational approach to link the variation of behavioural/trait markers with biological parameters. Such an approach might inform on biological processes that underly a spectrum spanning both non-clinical (non-diagnosed) and clinical subjects, yet it might be studied in different cohorts, including those lacking a diagnosis but displaying varying degrees of phenotype expression (including “subclinical” variation). In the present study, we aim to provide additional evidence for the association of subclinical traits / behaviours in individuals without a psychiatric condition with brain structural/functional parameters that are observed to be changed in previous case-control studies, and in our case specifically within the autistic spectrum.

Autism spectrum disorder (ASD) is a severe neurodevelopmental condition, defined by early-onset and persistent differences in social communication and the presence of restricted, repetitive, stereotyped behaviours and interests across multiple situations [8–10]. However, some of the phenotypic features identified in ASD individuals are also found in non-affected / non-autistic subjects in the general population, raising the possibility of an overlap or continuum of at least a part of the ASD phenotype. Recent psychometric and clinical findings suggest that autism spectrum disorders (ASD) might also be conceptualised as a combination of categorical and dimensional factors, as conceptualised in hybrid models [11, 12]. The latter might refer to and be conceptualised as behavioural traits, which can be quantified psychometrically, and are intrinsically related to ASD. For example, twin studies suggest moderate to high heritability of autistic traits, which were continuously distributed in a healthy control twin sample [13, 14]; this was particularly the case for the “Social Responsiveness Scale” [14]. Similarly, methylation studies imply shared epigenetics of ASD and autistic-like traits [15]. In addition, several recent studies have found correlations of autistic-like traits, such as those captured using the Autism Spectrum Quotient (AQ) and others, with regional brain volume variation in non-clinical samples, i.e., even in the absence of an ASD diagnosis [4, 16, 17].

While there is still an ongoing discussion whether ASD and its major symptoms and traits, as well as the putative broader autism phenotype [18], are best conceptualised in categorical, dimensional, or hybrid models [12, 19–21], these findings make a strong case for investigating the association of subclinical autistic traits and biological parameters. Similar to dimensional approaches within the clinical ASD spectrum (e.g., [22]), subclinical traits offer the opportunity to identify key brain structures and functional networks that may be related to clinically relevant phenotypes. For example, it is unclear whether autistic-like traits (either as a whole or distinct single facets) are related to brain networks overlapping with those identified in ASD case-control studies [23, 24] even though autistic-like traits may not always relate to ASD as they can relate to many other mental disorders [25].

In this present study, we build on previous studies that have identified associations of autistic-like traits in non-clinical populations with either volume-based brain structural parameters [4, 17] or structural connectivity inferred through diffusion tensor imaging (DTI) [5, 26]. While these studies have provided some important initial evidence of subclinical autistic-like trait phenotype correlates in the brain, they have some relevant shortcomings. First, they have focused on structural parameters that have a state-like component and are thus susceptible to additional (e.g., environmental) effects, such as the regional volume of a subcortical or neocortical region of interest. Secondly, not all of them have demonstrated how said structural associations might translate into functional effects (e.g., changes in activation or functional connectivity). We therefore devised a study design that would analyse cortical complexity, a more novel brain structural marker related to the folding of the neocortical surface. We chose cortical complexity as a marker of neocortical folding because it is assumed to reflect a parameter, which is rather static throughout most of adult life; it is thus assumed to be more indicative of early neurodevelopmental impacts, as opposed to cortical volume or thickness markers that might show subsequent changes or “compensation” [27, 28]. Cortical folding is most prominent during intrauterine brain development, probably extending to the first postnatal years, but then staying relatively stable over most of adult life [29, 30]. Hence, any variation detected in cortical folding in (early) adulthood is likely to reflect variation in early brain development in utero (or at least before age 4–6), and possibly related to genetic factors that impact on the formation of gyri. Recent findings show a moderate influence of (SNP-based) genetic risk for major psychiatric disorders on cortical complexity [31] as well as sensitivity to capture early brain development effects seen in pre-term born adult populations [32]. In addition to cortical folding parameters, we aimed to link any potential association of cortical complexity with autistic-like traits to variation in network connectivity using functional magnetic resonance imaging (fMRI). This approach links autistic-like traits, assumed to be associated with social interaction conditions like ASD, ADHD, mood or personality disorders [33–35] (van der Meer et al., 2012; van Steenel et al., 2013; May et al., 2021), with both early neurodevelopmental features and resulting variation in functional connectome parameters.

We tested the hypotheses that (a) autistic-like traits (as captured with the AQ) are association with variations in cortical complexity (a measure of early neurodevelopment) in non-clinical subjects, and (b) that the functional connectivity between such identified areas is related to cognitive-behavioural functional networks and altered with increasing autistic-like trait load / expression.

## Methods

### Subject cohort and phenotyping

We studied a cohort of *n* = 250 psychiatrically healthy subjects (173 female (69.2%) / 77 male (30.8%); mean age 23.9 yrs (SD = 3.9), average IQ of 116.87 (SD = 14.11), average handedness quotient (based on the Edinburgh Handedness Inventory, EHI [36]) of 79.75 (SD 52.75)) drawn from the general local population through advertisements and word-of-mouth recruitment, as used in a previous study [37]. All participants gave written informed consent to a study protocol approved by the Ethics Committee of the Medical School of Philipps-Universität Marburg (protocol number 61/18), in accordance with the latest version of the Declaration of Helsinki. Inclusion criteria included psychiatric health/well-being (validated with the SCID-I (structured clinical interview for DSM axis I, German version [38]) screening instrument), age 18–40 years and ability to provide informed consent and undergo an MRI scan; exclusion criteria included current or previous psychiatric conditions, traumatic brain injury, neurological (CNS) or uncontrolled general medical conditions potentially impacting on brain structure/function, or an intelligence quotient (IQ) of below 85, estimated with the multiple-choice vocabulary test (Mehrfachwahl-Wortschatz-Intelligenztest [39, 40]).

Resting state analyses were conducted in a slightly smaller subsample, consisting of 233 healthy individuals (161 female (69.1%), 72 male (30.9%) with an average age of 23.79 yrs (SD 3.8), an average IQ of 116.67 (SD 14.03) and an average handedness score of 79.89 (SD 52.84). This sample was slightly smaller owing to exclusion of 17 subjects for either missing data or quality control issues for resting state fMRI. Table 1 shows a detailed overview of the descriptive statistics for both samples.

**Table 1:** Descriptive statistics for the structural and resting state sample

	VBM sample			RS fMRI subsample		
	*N*	mean (SD)	range	*N*	mean (SD)	range
Sex	173 f / 77 m	-	-	161 f/ 72 m	-	-
Age	250	23.90 (3.92)	18–40	233	23.79 (3.84)	18–39
IQ	250	116.87 (14.11)	93–145	233	116.67 (14.03)	93–145
EHI LQ	250	79.75 (52.75)	-100-100	233	79.89 (52.84)	-100-100
AQ Total	250	13.14 (5.01)	2–36	233	13.15 (5.02)	4–36
AQ Social Skills	250	2.37 (1.63)	0–10	233	2.33 (1.52)	0–9
AQ Attention Switching	250	3.77 (1.78)	0–9	233	3.72 (1.71)	0–9
AQ Attention to Detail	250	3.69 (2.14)	0–10	233	3.67 (2.11)	0–10
AQ Communication	250	1.53 (1.38)	0–7	233	1.54 (1.40)	0–7
AQ Imagination	250	1.88 (1.65)	0–9	233	1.89 (1.66)	0–9

SD = standard deviation; IQ = intelligence quotient, estimated with the multiple-choice vocabulary test (Mehrfachwahl-Wortschatz-Intelligenztest, MWTB; [39]); EHI LQ = Edinburgh Handedness inventory laterality quotient,

### Phenotyping of autistic-like traits using AQ (Autism Questionnaire)

As described in our previous study [37], we used the Autism Spectrum Quotient (AQ) to characterise our sample for autistic-like traits. The AQ, a self-administered questionnaire used to measure autistic-like traits in adults [41] has been used widely in a number of clinical and non-clinical studies (meta-analysis in [42]). While the use of AQ in screening for ASD has been discussed, particularly in comparison with other measures [43], the large body of data in non-clinical healthy adults [42] makes it a well-established tool for measuring autistic-like traits in non-clinical populations, including identification of individuals with higher levels of social problems in clinical settings. It has also been widely used for differential comparison with other phenotypes ( [19, 44]) and general population samples [41, 45].

The AQ provides a total score of autistic-like traits, and five subscores, i.e., attention to detail, attention switching, communication, imagination, and social skills. As described previously for this cohort, AQ subscales show satisfactory internal consistency, except for low Cronbach’s alpha in the AQ attention to detail subscale (see inter-item correlations in [4]).

Subjects completed the AQ within a week of MRI scanning using a secured electronic platform, checking for completeness of obtained data.

### Magnetic resonance imaging (MRI): data acquisition and pre-processing

We obtained structural and functional MRI data sets on a 3 Tesla scanner (Magnetom Tim Trio, Siemens Healthineers, Erlangen, Germany) with a 12-channel dedicated head matrix Rx-coil.

For structural MRI analysis, we used T1-weighted high-resolution anatomical scans from a three-dimensional MPRAGE sequence with gradient echo, an acquisition time of 4:26 min and the following parameters: repetition time (TR) = 1900ms, echo time (TE) = 2.26ms, inversion time (TI) = 90ms, field-of-view (FoV) = 256 mm, flip angle (FA) = 9°. We acquired 176 slices an isotropic resolution of 1 × 1 × 1mm^3^.

For functional MRI analysis, we used a resting-state T2*-weighted echo planar imaging (EPI) protocol, which involved subjects to be instructed to rest for 8 min with eyes closed, and room lights turned off. EPI acquisitions used the following sequence parameters: TR = 2000ms, TE = 30ms, FoV = 210 × 210mm^2^, 33 slices with a slice thickness of 3.8 mm and an in-plane resolution of 3.3 × 3.3mm^2^.

### Cortical complexity analysis

We used the CAT12 toolbox (version rs1720) for pre-processing and analysis of cortical complexity data, applying the recommended default options (Computational Anatomy Toolbox 12, C. Gaser, Structural Brain Mapping Group, University of Jena, Germany).

The cortical complexity measure implemented in CAT12 is based on spherical harmonics modelling of the cortical surface using a fractal dimensions approach [46]. Surface reconstruction includes topology correction and spherical registration [46]. Cortical complexity images were smoothed with a Gaussian kernel of 20 mm FWHM (full width at half maximum).

Previous studies have used the cortical complexity measure to identify cortical folding abnormalities in neuropsychiatric disorders such as schizophrenia [46, 47] and bipolar disorder [48].

### Functional brain connectivity analysis

Based on the findings derived from cortical complexity analyses, we performed an analysis of functional connectivity to link the correlation of cortical complexity with AQ scores to potential functional effects.

We used the CONN tool box [49] for pre-processing and time-series analysis of resting-state fMRI scans. Using the default pre-processing pipeline, images were realigned using the SPM12 realign & unwarp procedure [50], co-registered and resampled to a reference image, and normalised into standard MNI space. Images were smoothed with a Gaussian kernel of 4 mm FWHM and denoised with the default denoising pipeline [51]. After denoising, structural and functional normalised images were visually inspected for artefacts and normalisation failures. Further, individuals with less than 75% valid scans (according to the automatic CONN detection algorithm) were excluded (*n* = 2), leading to the final sample of *n* = 233.

### Statistical analysis

Our hypotheses were tested in two steps. First, we used vertex-wise maps of cortical complexity across the entire neocortex to set up general linear models (GLMs), separately for AQ-total scores and each AQ subscore, with age and sex as covariates, in order to compute vertex-wise correlations between cortical complexity and each AQ subscore. Separate GLMs were used since AQ subscores are often at least moderately inter-correlated with each other, and inclusion of multiple AQ scores in a single GLM thus would have removed variance distorting structure-trait variations. We applied the cluster-level FWE (family-wise error) approach with *p* < 0.05 thresholding (*p* < 0.001 uncorrected for cluster definition) to correct for multiple-comparisons. We performed a supplementary analysis including IQ as a nuisance variable in the GLM in order to remove variance related to differences in IQ. IQ was not correlated with AQ *total* or any of the subscores (AQ total *r*=-0.07, *p* = 0.245; AQ Social Skills *r* = 0.02, *p* = 0.695; AQ Attention Switching *r*=-0.05, *p* = 0.459; AQ Attention to Detail *r*=-0.07, *p* = 0.262; AQ Communication *r*=-0.06, *p* = 0.314; AQ Imagination *r*=-0.05, *p* = 0.426).

Second, we performed a seed-based functional connectivity analysis of resting-state fMRI data. Based on our cortical folding complexity findings, we identified matching regions from the Glasser360 atlas [52], specifically the left and right ventral posterior part of the superior temporal sulcus (STSvp), which were defined as seeds. Using the CONN toolbox and its statistical module, we then applied regression models correcting for age and sex and tested for significant associations of the AQ scores and functional connectivity between each of the seeds with each voxel in the brain. Applying standard setting for cluster-based inferences (random field theory parametric statistics), we again used cluster-level FWE correction to account for multiple comparisons of brain-wide analysis. Again, we performed an additional supplementary analysis correcting for IQ (including IQ as a nuisance variable in the GLM). We corrected for age and sex in all of the GLM analyses of both cortical complexity and resting-state functional connectivity.

## Results

### Cortical complexity analyses

**Fig. 1:**
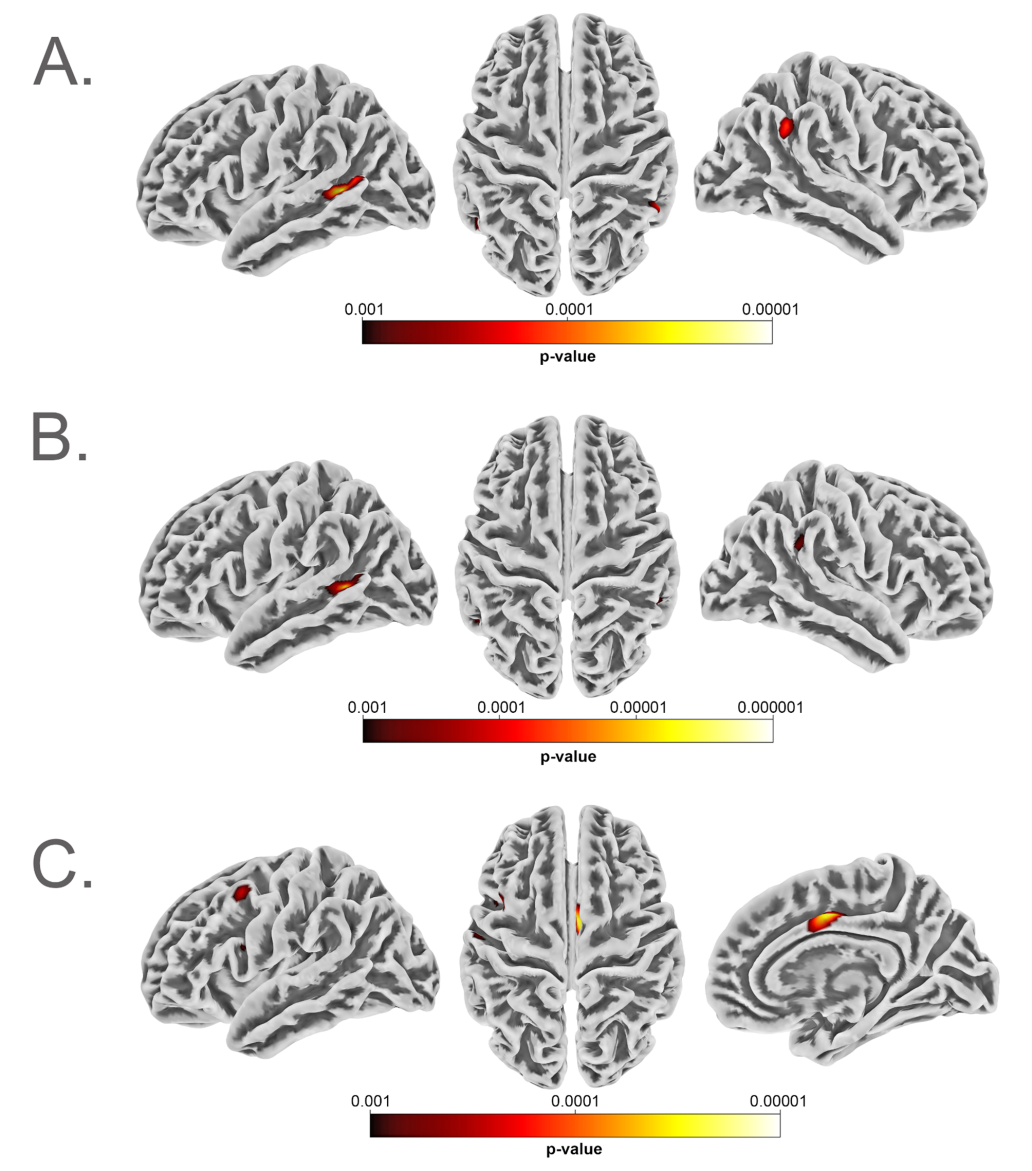
Correlation of cortical surface complexity (calculated using CAT12 software) and AQ total score (**A**; significant negative correlation, maximum intensity voxel at co-ordinates x/y/z: -51/-49/+5; *p* = 0.009); AQ Attention Switching subscore (**B**; significant negative correlation, maximum intensity voxel at co-ordinates x/y/z: -53/-51/+4); *p* = 0.013; and AQ communication (**C**; significant positive correlation, maximum intensity voxel at co-ordinates x/y/z: +6/-5/+42; *p* = 0.043) resp.; images are thresholded at *p* < 0.001 uncorrected

We found a negative correlation between *AQ total score* and cortical complexity in the left superior temporal sulcus (ROI: bankssts) (*p* = 0.009 FWE cluster-level corrected, 190 voxels), as shown in Fig. 1. Higher AQ attention switching scores also showed a negative correlation with cortical complexity in the left superior temporal sulcus. (*p* = 0.013 FWE cluster-level corrected, 177 voxels).

A positive correlation was found for AQ communication scores and cortical complexity in the right posterior cingulate (*p* = 0.043, FWE cluster-level corrected, 134 voxels).

An overview of significant clusters for cortical complexity analyses providing co-ordinates, significance values and effect size parameters is given in Table 2.

**Table 2:** Significant clusters in the correlation analyses of cortical surface complexity and AQ scores

subscale	correlation	*r*	k	*p*	x/y/z
AQ total score	negative	-0.258	190	0.009	-51/-49/+05
AQ attention switching	negative	-0.271	177	0.013	-53/-51/+04
AQ communication	positive	0.257	134	0.043	+ 06/-05/+42

r = correlation strength at peak vertex; k = number of voxels in the cluster; *p* = FWE cluster-level corrected significance value; x/y/z = peak coordinates of the cluster

Supplementary analyses correcting for IQ showed almost identical findings (see Supplementary Fig. 1 and Supplementary Table 1 for details).

### Functional connectivity analyses

We found a significant negative correlation between the AQ total score and functional connectivity between the right STSvp and a cluster in the left lateral occipital cortex (k = 70 voxels, x/y/z=-38/-60/+44, *p* = 0.023, FWE cluster-level-corrected), apparently driven by the AQ attention to detail subscale that showed a negative correlation to a very similar cluster (k = 67 voxels, x/y/z=-36/-60/+42, *p* = 0.029, FWE cluster-level-corrected) with the right STSvp as seed.

We found a positive correlation between the AQ attention switching subscale and functional connectivity between the left STSvp and a cluster in the left inferior/middle frontal gyrus (k = 84 voxels, x/y/z=-50/+28/+20, *p* = 0.009, FWE cluster-level-corrected).

**Fig. 2:**
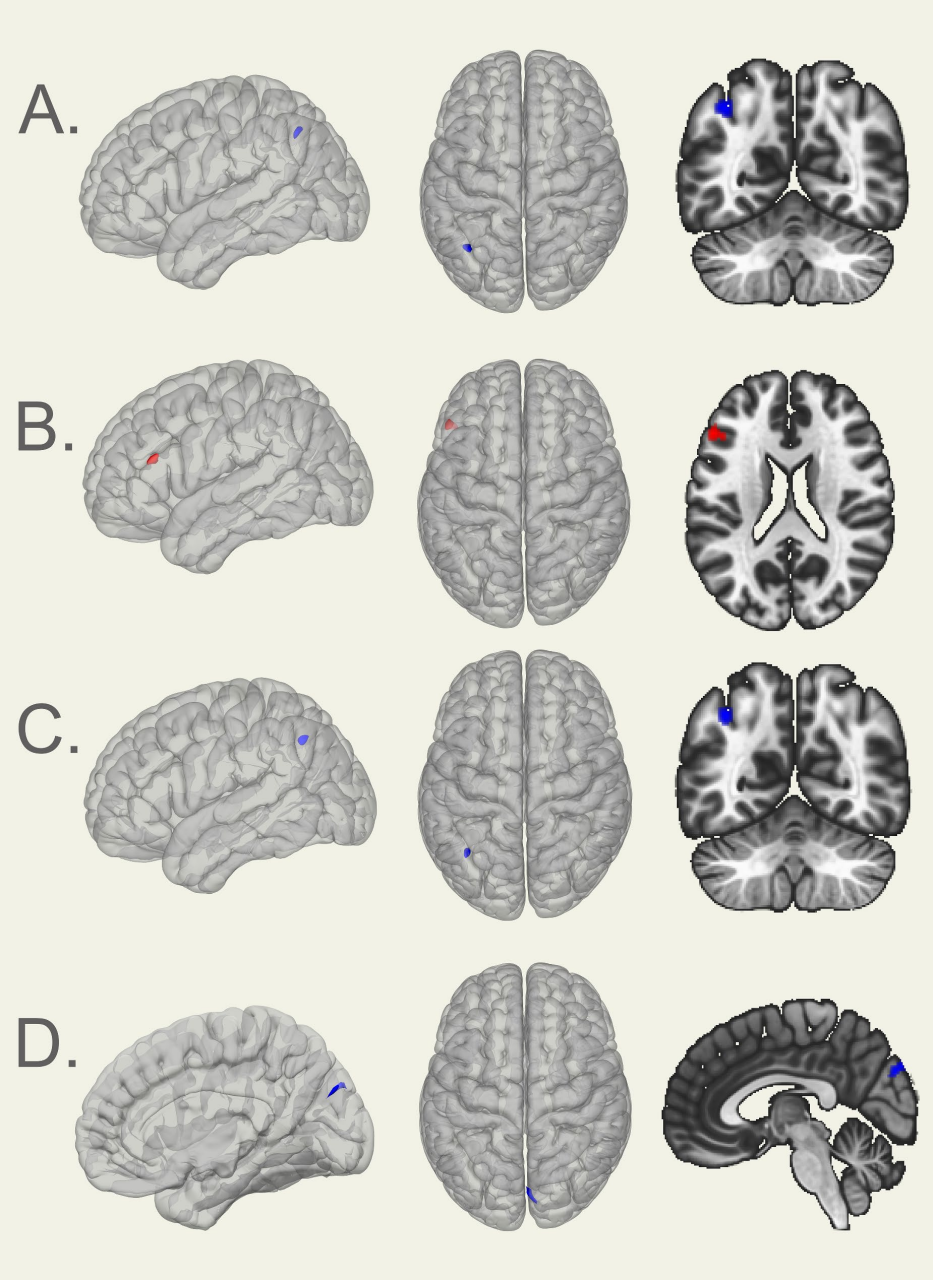
Significant clusters in the resting state seed to voxel analysis with right and left STSvp as seeds. (**A**) AQ Total (negative correlation, maximum intensity voxel at co-ordinates x/y/z: -38/-60/+44; *p* = 0.023), (**B**) AQ Attention Switching (positive correlation, maximum intensity voxel at co-ordinates x/y/z: -50/+28/+20; *p* = 0.009) (**C**) AQ Attention to Detail (negative correlation, maximum intensity cluster at co-ordinates x/y/z: -36/-60/+42; *p* = 0.029) (**D**) AQ Communication (negative correlation, maximum intensity cluster at co-ordinates x/y/z: +4/-80/+32; *p* = 0.018)

Lastly, we found a negative correlation between the AQ communication subscale and functional connectivity between the left STSvp and a cluster in the right cuneal cortex (k = 74 voxels, x/y/z = + 4/-80/+32, *p* = 0.018, FWE cluster-level-corrected). Figure 2 shows the significant clusters obtained from the resting-state analysis. An overview of significant clusters for functional connectivity analyses providing co-ordinates, significance values and effect size parameters is given in Table 3.

**Table 3:** Significant clusters in the resting state seed-to-voxel analysis for the respective AQ scores, with left and right STSvp as seed region

subscale	seed region	correlation	*r*	k	*p*	x/y/z
AQ total score	right STSvp	negative	-0.079	70	0.023	-38/-60/+44
AQ attention switching	left STSvp	positive	0.084	84	0.009	-50/+28/+20
AQ attention to detail	right STSvp	negative	-0.081	67	0.029	-36/-60/+42
AQ communication	left STSvp	negative	-0.086	74	0.018	+ 4/-80/+32

r = correlation strength; k = number of voxels in the cluster; *p* = FWE cluster-level corrected significance value; x/y/z = peak coordinates of the cluster

Again, supplementary analyses including correction for IQ showed mostly similar findings to the main analyses (see Supplementary Fig. 2 and Supplementary Table 2 for details).

## Discussion

Subclinical expression of disease-related phenotypes might offer relevant insights into the neurobiological basis of disease spectra, while studying non-clinical cohorts avoids confounding factors such as the effects of psychotropic medication or co-morbidities typically encountered in case-control designs [24]. As this study set out to identify dimensional neural correlates of autistic-like traits in non-clinical subjects, our analyses point to three major findings: first, cortical folding in the left superior temporal sulcus being associated with both AQ total scores and particularly the attention switching subscale; second, changes of functional connectivity from the left STSvp to other brain areas with increasing autistic-like traits, and third, a dissociation of functional connectivity patterns and overall autistic-like traits (AQ total) vs. attention switching AQ traits, where overall autistic-like traits predict functional connectivity of left STS with occipital cortices, but AQ attention switching is related to functional connectivity of the left STS to left lateral prefrontal cortex.

### Association of cortical complexity with autistic-like traits

Measures of cortical gyrification capture morphometric brain features different from more conventional parameters such as regional volumes or cortical thickness. Recent basic science findings have added to the general understanding of cortical development [53], but more specifically to the mechanics of cortical folding (e.g. [54]), and the heterochronicity of that process across different cortical areas [55]. On the cellular level, transcriptomics studies have shown that mid-gestational expression and cell differentiation is regulated by genes implicated in ASD [56], while primate studies link genes implicated in ASD to additional postnatal disease-related gene expression [57].

Our gyrification finding implies that subtle variations in this highly developmental parameter can be linked to subclinical autistic-like traits even in the absence of clinical conditions, thus questioning an interpretation of previous ASD case-control studies (e.g., [58, 59]), relating gyrification to the developmental nature of ASD. Rather, at least a part of that variance in gyrification could be related to either an extended phenotype or a dimensional phenotype that spans far beyond clinical and subclinical forms of problems in social interaction. In contrast to most MR morphometric studies in ASD case-control designs (e.g [24, 60, 61]). as well as to more recent dimensional association studies of autistic-like traits in non-clinical subjects (e.g [4, 62]). or mixed samples [63], gyrification is linked to a complex early development of the cortical folds. Our use of cortical complexity as a measure of neocortical folding puts to use a somewhat more recently developed method, which applies a spherical harmonics approach and therefore complements earlier measures of cortical folding.

Three recent studies, although using approaches markedly different from our analysis, merit attention and comparison to our findings: First, a recent study [64] in children and adolescents age 13–16 years has linked gyrification to recent autistic-like traits in a non-autistic cohort (using the parent-assessed Social Responsiveness Scale, SRS) to frontal and temporal areas, including parts of the superior temporal gyrus (more so in the right hemisphere); a second study by Alemany et al. showed in a large cohort of children that autistic-like traits (but not polygenic risk for ASD) are associated with parietal and lateral occipital gyrification [65]. Finally, a study using the Autism Diagnostic Observation Schedule (ADOS-2) showed an association of autistic-like traits, albeit in eating disordered underweight individuals rather than ASD or non-clinical subjects, with gyrification in the postcentral and supramarginal cortices [66]; given the potential effects of weight loss not only on brain water content but possibly also other metrics limits comparability with other studies.

While case-control studies have not consistently shown gyrification abnormalities in ASD (e.g. [67]), more recent stratification within ASD individuals indicates that subgroups with “reciprocal social interaction” and “restricted, repetitive, and stereotyped patterns of behavior” do show increased gyrification [59], echoing previous studies on structural connectivity and repetitive behaviour in ASD [68].

### Implications of functional connectivity associations with autistic-like traits

While our gyrification findings are consistent with a (quasi-)dimensional brain structural correlate of autistic-like traits functional connectivity analysis supports this on a functional level. The involvement of posterior STS in social (dys)function and ASD has been shown in previous behavioural studies (e.g. reviews in [69, 70]), as well as meta-analysis of fMRI activation studies across clinical cohorts [71]. While there are few previous studies on fMRI, particularly analyses of functional connectivity, and autistic-like traits in clinically healthy cohorts, our findings share similarities with both a growing number of studies in autism-like traits and task-based fMRI in “neurotypical” subjects. For example, in a cohort of 30 clinically healthy subjects, AQ predicted functional coupling of pSTS activation during eye contact and gaze patterns with the fusiform face area [72] and in another study between STS and dorsomedial prefrontal cortices during mentalisation efforts [73], although aberrant STS connectivity has also been observed in non-visual tasks in non-clinical subjects with autistic-like features [74].

A noteworthy aspect of our functional connectivity findings is the dissociation between connectivity patterns and subscales of the AQ, correlations between pSTS and other brain areas depended on the AQ subscale with significant correlations towards the lateral occipital cortex for the AQ attention to detail subscale, towards the inferior/middle frontal gyrus for AQ attention switching, and towards the cuneus for AQ communication. This suggests that a summary autistic trait score might be limited in detecting associations for particular facets of autism-like traits (see also [75]). This also has implications for interpreting case-control studies, in which categorical comparison is frequently made without considering the heterogeneity within clinical cohorts (i.e., the case-control comparison relies on the overall sum of symptoms/traits, rather than single facets or subscores that might be more useful for cohort stratification or association with brain mechanisms). A recent case control study covering a larger age range of children and adults (age 7–30 years) has suggested that age might be an important factor as connectivities of the pSTS might depend on maturation of underlying circuits [76]. While the age range of our sample precludes direct replication, it should be pointed out that the former findings might rather be driven by the developmental heterogeneity of the ASD cohort.

Different facets autistic-like traits might link to the pSTS because of its unique involvement in multiple brain networks subserving functions that range from socially relevant higher visual integration to “theory of mind” functions [77], emotion recognition [78], and reward functions [79], as well as because of its connections with brain regions involved in attention to visual detail (through its connection with the lateral occipital cortex) and reorienting of visual attention from the current focus towards new stimuli (through its connection with the inferior frontal gyrus) [80, 81].

## Limitations

While our study provides several findings that confirm a linear relationship between autistic-like traits in non-clinical subjects and both gyrification (as an indicator of early neurodevelopment) and functional connectivity of posterior STS, there are some limitations to be considered. First, we applied the AQ for assessment of autistic-like traits; although widely used in non-autistic cohorts [42], some subscores have received criticism in recent psychometric studies [75]. Since we did not obtain additional measures of autistic-like traits, replications with additional / complementary inventories as well as studies in well characterised ASD samples to further examine the assumed dimensionality would be desirable. Second, our cohort demographic shows differences to age and IQ distributions typically observed in ASD cohorts [10], which needs to be taken into account. Third, our study did not collect behavioural data that would have allowed us to directly link structural or functional parameters to functions such as eye gaze or social interaction. Despite the consistency with pSTS studies in people with ASD and other psychiatric conditions [77, 82], larger combined study cohorts would be desirable and needed. While we acknowledge recent studies highlighting the difficulties of identifying robust and replicable brain-phenotype correlations [83], it is worth pointing out that global psychopathological markers, such as used in the mentioned study, might be less suitable compared to more specific phenotype facets as given with the AQ subscales (despite some psychometric imperfections, see ). In overcoming the difficulties of insufficient power of brain-behaviour association studies [84], it has been argued that such fine-grained phenotyping might be essential in overcoming the limitations of limited replicability in previous study designs [85].

## Conclusions

In conclusion, our study supports a dimensional relationship between the expression of autistic-like traits in the non-clinical spectrum with brain structural variation, in particular cortical folding of the posterior STS, a key region for perception integration and social functioning. The pSTS functional connectivity during rest is furthermore associated with different occipital and frontal brain areas, in dependence of phenotype sub-facets, which highlights the importance of relating particular discernible aspects of a broader autism phenotype to specific circuitry in dimensional models of psychopathology [86].

## Electronic supplementary material

Below is the link to the electronic supplementary material.

Supplementary Material 1

## Data Availability

Original data can be made available upon reasonable request and pending local and national data protection and ethics regulation.
