# Supplementary material for: Superior temporal sulcus folding, functional network connectivity, and autistic-like traits in a non-clinical population
Source: Mol Autism. 2024 Oct 8;15:44. doi: 10.1186/s13229-024-00623-3 (PMC11463051; doi:10.1186/s13229-024-00623-3)

**Supplementary Table 1**

Significant clusters in the correlation analyses of cortical surface complexity and AQ scores with and without correction for IQ.

| **subscale** | **correlation** | **k** | **p** | x/y/z |
| --- | --- | --- | --- | --- |
| AQ total score | negative | 190 | 0.009 | -51/-49/+05 |
| AQ total score with correction for IQ | negative | 176 | 0.014 | -51/-49/+05 |
| AQ attention switching | negative | 177 | 0.013 | -53/-51/+04 |
| AQ attention switching with correction for IQ | negative | 170 | 0.016 | -53/-51/+04 |
| AQ communication | positive | 134 | 0.043 | +06/-05/+42 |
| AQ communication with correction for IQ | positive | 133 | 0.044 | +06/-05/+42 |

k = number of voxels in the cluster; p = FWE cluster-level corrected significance value; x/y/z = peak coordinates of the cluster

**Supplementary Table 2**

Significant clusters in the resting state seed-to-voxel analysis for the respective AQ scores, with left and right STSvp as seed region with and without correction for IQ.

| **subscale** | **seed region** | **correlation** | **k** | **p** | x/y/z |
| --- | --- | --- | --- | --- | --- |
| AQ total score | right STSvp | negative | 70 | 0.023 | -38/-60/+44 |
| AQ total score with correction for IQ | right STSvp | negative | 75 | 0.016 | -38/-60/+44 |
| AQ attention switching | left STSvp | positive | 84 | 0.009 | -50/+28/+20 |
| AQ attention switching with correction for IQ | left STSvp | positive | 84 | 0.009 | -50/+26/+22 |
| AQ attention to detail | right STSvp | negative | 67 | 0.029 | -36/-60/+42 |
| AQ attention to detail with correction for IQ | right STSvp | negative | 65 | 0.034 | -36/-60/+42 |
| AQ communication | left STSvp | negative | 74 | 0.018 | +04/-80/+32 |
| AQ communication (with correction for IQ) | left STSvp | negative | 64 | 0.037 | +04/-80/+32 |

k = number of voxels in the cluster; p = FWE cluster-level corrected significance value; x/y/z = peak coordinates of the cluster

**Supplementary Figure 1**

Correlation of cortical surface complexity (calculated using CAT12 software) and AQ total score (A.; negative correlation, maximum intensity voxel at co-ordinates x/y/z: -51/-49/+5; p=0.014); AQ Attention Switching subscore (B.; negative correlation, maximum intensity voxel at co-ordinates x/y/z: -53/-51/+4); p=0.016; and AQ communication (C.; positive correlation, maximum intensity voxel at co-ordinates x/y/z: +6/-5/+42; p=0.044) resp.; images are thresholded at p<0.001 uncorrected


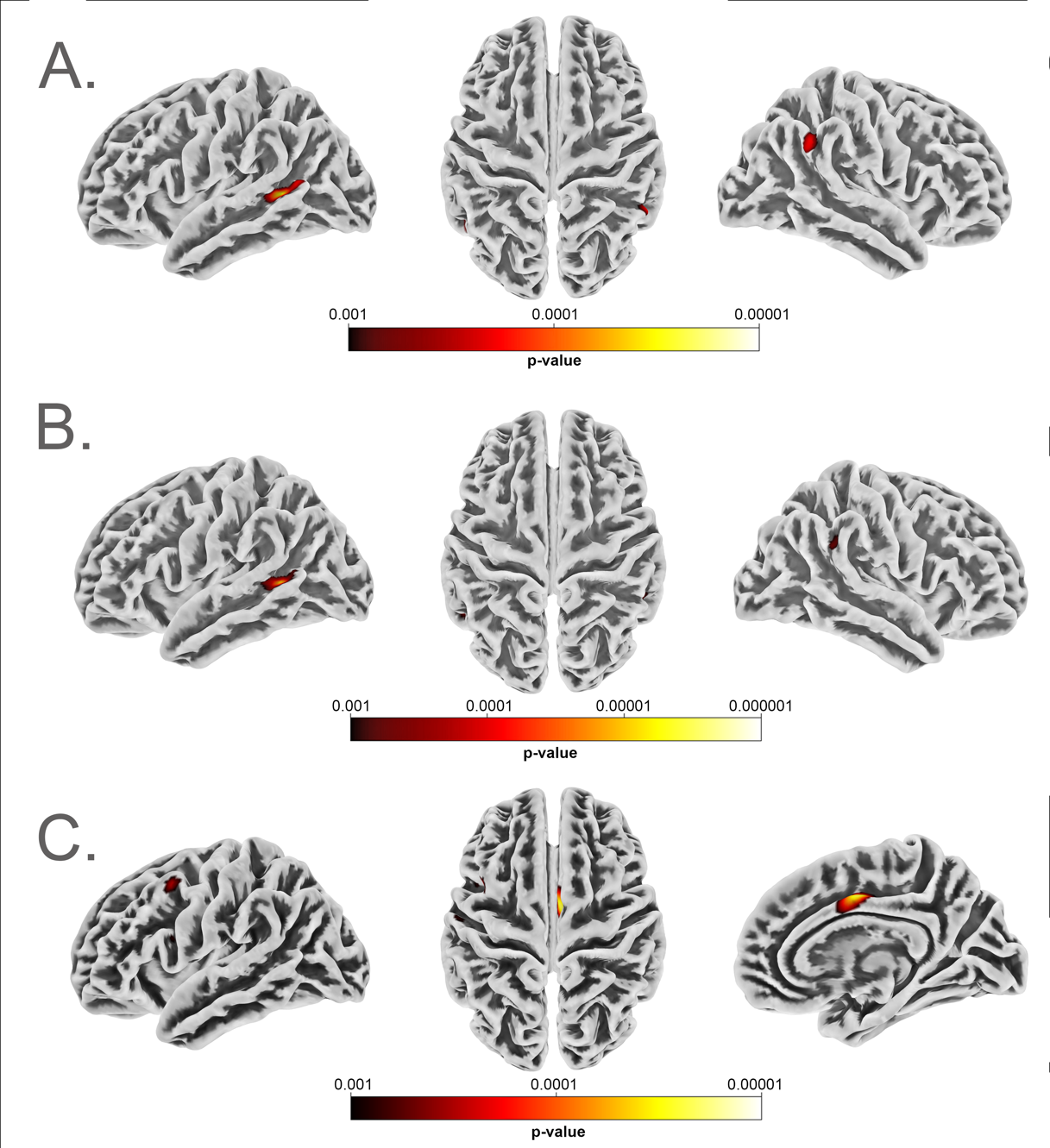


**Supplementary Figure 2**

Significant clusters in the resting state seed to voxel analysis with right and left STSvp as seeds. A. AQ Total (negative correlation, maximum intensity voxel at co-ordinates x/y/z: -38/-60/+44; p=0.016), B. AQ Attention Switching (positive correlation, maximum intensity voxel at co-ordinates x/y/z: -50/+26/+22; p=0.009) C. AQ Attention to Detail (negative correlation, maximum intensity cluster at co-ordinates x/y/z: -36/-60/+42; p=0.034) D. AQ Communication (negative correlation, maximum intensity cluster at co-ordinates x/y/z: +4/-80/+32; p=0.037).


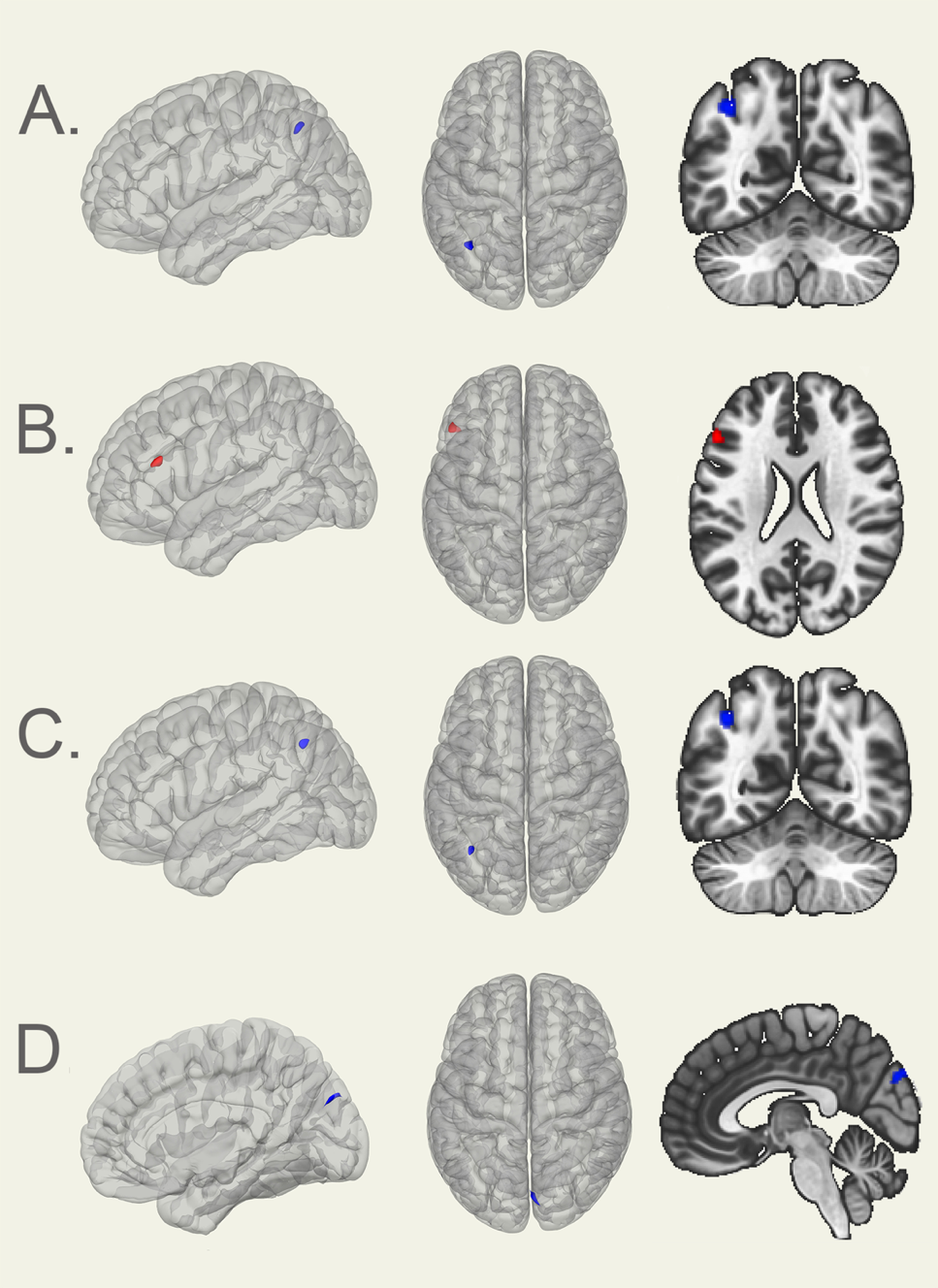

Supplement: Supplementary file 1 — Supplementary Material 1 [file 13229_2024_623_MOESM1_ESM.docx]
